# Mass Spectrometry-Based Proteomics for Investigating DNA Damage-Associated Protein Ubiquitylation

**DOI:** 10.3389/fgene.2016.00109

**Published:** 2016-06-14

**Authors:** Jan B. Heidelberger, Sebastian A. Wagner, Petra Beli

**Affiliations:** ^1^Institute of Molecular BiologyMainz, Germany; ^2^Department of Medicine, Hematology/Oncology, Goethe UniversityFrankfurt, Germany

**Keywords:** mass spectrometry-based proteomics, ubiquitin, ubiquitin-modifying enzymes, ubiquitin remnant profiling, DNA damage response

## Abstract

Modification of proteins with the 76 amino acid protein ubiquitin plays essential roles in cellular signaling. Development of methods for specific enrichment of ubiquitin remnant peptides and advances in high-resolution mass spectrometry have enabled proteome-wide identification of endogenous ubiquitylation sites. Moreover, ubiquitin remnant profiling has emerged as a powerful approach for investigating changes in protein ubiquitylation in response to cellular perturbations, such as DNA damage, as well as for identification of substrates of ubiquitin-modifying enzymes. Despite these advances, interrogation of ubiquitin chain topologies on substrate proteins remains a challenging task. Here, we describe mass spectrometry-based approaches for quantitative analyses of site-specific protein ubiquitylation and highlight recent studies that employed these methods for investigation of ubiquitylation in the context of the cellular DNA damage response. Furthermore, we provide an overview of experimental strategies for probing ubiquitin chain topologies on proteins and discuss how these methods can be applied to analyze functions of ubiquitylation in the DNA damage response.

## Mass Spectrometry-Based Proteomics for Investigating Posttranslational Modifications

Mass spectrometry (MS)-based proteomics has become a powerful tool for investigating posttranslational modifications (PTMs) of proteins in the context of cellular signaling (Larance and Lamond, 2015). In shotgun or bottom up proteomics, cellular proteins are subjected to proteolysis and the resulting peptides are separated according to hydrophobicity using high-pressure liquid chromatography (LC) and identified by tandem mass spectrometry (LC–MS/MS; Aebersold and Mann, 2003). The mass difference introduced by the presence of PTMs can be exploited for identification and localization of modifications. A major obstacle for proteome-wide analysis of posttranslational modification sites by LC–MS/MS is the sub-stoichiometric cellular occurrence of modified protein species and the inability of current mass spectrometers to identify all peptides resulting from digestion of the cellular proteome. Consequently, specific enrichment methods for modified peptide species are essential for the proteome-wide identification of PTMs by LC-MS/MS.

Identification of endogenous ubiquitylation sites by MS-based proteomics has long been hampered by a lack of specific enrichment methods for ubiquitylated peptides. Earlier studies have most commonly relied on the ectopic expression of polyhistidine tagged ubiquitin and enrichment of ubiquitylated proteins by nickel-nitrilotriacetic acid (Ni-NTA) chromatography under denaturing conditions. Alternatively, purified tagged ubiquitin-binding domains have been used to enrich ubiquitylated proteins from cells expressing endogenous ubiquitin (Hjerpe et al., 2009). These approaches have been successfully employed for the identification of putative ubiquitylated proteins and a limited number of ubiquitylation sites (Peng et al., 2003; Matsumoto et al., 2005; Tagwerker et al., 2006; Meierhofer et al., 2008; Danielsen et al., 2011). However, enrichment at the protein level does not sufficiently reduce sample complexity and thus does not permit efficient proteome-wide identification of endogenous ubiquitylation sites.

## Ubiquitin Remnant Profiling for Proteome-Wide Identification of Ubiquitylation Sites

**FIGURE 1:**
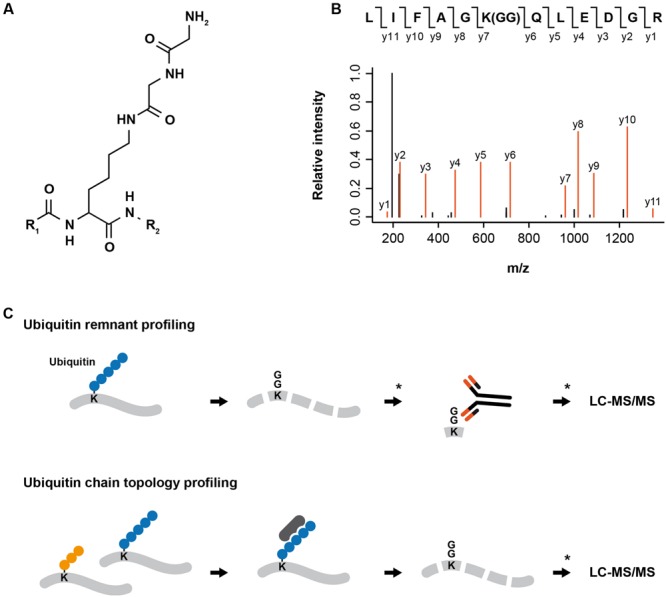
**MS-based proteomics approaches for analyzing protein ubiquitylation. (A)** Digestion of ubiquitylated proteins with trypsin leaves a di-glycine remnant from the C-terminus of ubiquitin covalently attached to the previously modified lysine. **(B)** Exemplary fragment spectrum of a di-glycine modified peptide. The di-glycine remnant leads to a shift of ∼114 Da in the peptide mass and can be exploited to pinpoint the localization of the ubiquitin attachment. **(C)** For ubiquitin remnant profiling, proteins extracted from cells are digested into peptides using trypsin, di-glycine modified peptides are enriched using di-glycine lysine specific antibodies and identified by LC-MS/MS. ^∗^Pre- and post-enrichment fractionation can be introduced to decrease sample complexity and increase the depth of the analysis. For ubiquitin chain topology profiling, proteins extracted from cells or tissues are incubated with an ubiquitin linkage-specific binder (e.g., antibody, affimer, TUBE). Enriched proteins modified by a specific type of ubiquitin chain are digested in-gel into peptides and peptide samples are analyzed by LC-MS/MS. ^∗^Post-enrichment fractionation can be introduced to decrease sample complexity and increase the depth of the analysis.

Digestion of ubiquitylated proteins with trypsin leaves a di-glycine remnant from the C-terminus of ubiquitin covalently attached to the previously ubiquitylated lysine. The di-glycine remnant leads to a peptide mass shift (∼114 Da) and can be exploited to pinpoint the localization of the ubiquitin attachment site in the protein (Peng et al., 2003) (**Figure 1**). Xu et al. (2010) used di-glycine-modified histones as an antigen to produce the monoclonal antibody GX41 that specifically recognizes di-glycine adducts on the 𝜀-amine of lysine. To demonstrate the applicability of the antibody for enrichment of di-glycine modified peptides, the authors purified ubiquitylated proteins from HEK293T cells ectopically expressing His_6_-tagged ubiquitin. After proteolysis, di-glycine-modified peptides were enriched using di-glycine lysine specific antibodies and identified by LC–MS/MS.

The generation of di-glycine lysine specific antibodies and advances in high-resolution mass spectrometry have enabled first proteome-wide studies of endogenous ubiquitylation sites in human cells (Kim et al., 2011; Wagner et al., 2011) (**Figure 1**). Wagner et al. (2012) extended ubiquitin remnant profiling to murine tissues and demonstrated that different tissues display specific ubiquitylation patterns. In addition, they identified a number of core ubiquitylation sites that are present in all examined tissues.

One drawback of ubiquitin remnant profiling is that tryptic digestion of proteins modified with the ubiquitin-like modifiers NEDD8 and ISG15 also results in a di-glycine remnant attached to the previously modified lysine. Consequently, ubiquitin remnant profiling does not allow distinguishing NEDD8- and ISG15-modification sites from ubiquitylation sites. However, the expression of ISG15 in cells that are not stimulated with interferon (IFN)-α/β is very low (Skaug and Chen, 2010) and NEDD8 is considered to primarily modify Cullin-RING ligases (CRLs; Lydeard et al., 2013). Kim et al. (2011) used different experimental approaches to demonstrate that >94% of the di-glycine lysine containing peptides are indeed ubiquitin remnant peptides.

## Quantitative Analyses of Protein Ubiquitylation By Ubiquitin Remnant Profiling

Ubiquitin remnant profiling can be combined with quantitative proteomics approaches based on metabolic (e.g., stable isotope labeling with amino acids in cell culture, SILAC) or chemical labeling (e.g., tandem mass tags or isobaric tags for relative and absolute quantitation) to quantify the relative abundance of ubiquitylation sites in different experimental conditions. In the last 5 years this strategy has been successfully implemented to investigate site-specific alterations of the ubiquitin-modified proteome after cellular stress or growth factor stimulation as well as to identify substrates of ubiquitin-modifying enzymes.

Initial studies employed ubiquitin remnant profiling and SILAC-based quantitative proteomics to analyze site-specific changes in ubiquitylation in different human cell lines after inhibition of the proteasome (Kim et al., 2011; Wagner et al., 2011; Udeshi et al., 2012). These studies have shown that proteasome inhibition globally perturbs cellular ubiquitylation patterns and leads to increased ubiquitylation of >40% of the quantified sites. Interestingly, proteasome inhibition also results in decreased abundance of a fraction of ubiquitylation sites, including sites on histones and DNA repair factors PCNA, FANCI, and FANCD2 that confer non-degradative, regulatory functions. These observations indicate that proteasome inhibition leads to depletion of the cellular ubiquitin pool and results in a shift from mono- to poly-ubiquitylation, and can therefore be used to distinguish degradative from non-degradative, regulatory ubiquitylation (Kim et al., 2011; Wagner et al., 2011; Udeshi et al., 2012; Higgins et al., 2015).

Ubiquitin remnant profiling and SILAC-based quantitative proteomics has been used to perform first proteome-wide, site-specific analyses of ubiquitylation after DNA damage. Povlsen et al. (2012) induced DNA damage in U2OS cells by ultraviolet light (UV) irradiation and identified up-regulated and down-regulated ubiquitylation sites on known components of DNA damage repair and signaling as well as on proteins that had not been previously implicated in this process, demonstrating that ubiquitin-modifying enzymes play an integral role in the regulation of the cellular response to DNA damage. This study also demonstrated that UV light affects the ubiquitylation status of the PCNA-associated factor PAF15 and that PAF15 ubiquitylation regulates the interaction between translesion synthesis polymerases and PCNA during the DNA damage bypass. Another study investigated site-specific changes in ubiquitylation after DNA damage induced by irradiation of U2OS cells with UV light or ionizing radiation (IR) and could demonstrate that centromere proteins are de-ubiquitylated in response to UV light- and IR-induced DNA damage (Elia et al., 2015a). Notably, in this study profiling of ubiquitylation sites has been performed with and without pre-treatment of cells with the proteasome inhibitor MG132 to facilitate the identification of both degradative and non-degradative, regulatory ubiquitylation sites, respectively. In another study, the same authors found that the single-stranded DNA binding protein RPA is ubiquitylated on multiple lysines after replication fork stalling by the ubiquitin ligase RFWD3 (Elia et al., 2015b). Ubiquitylation of RPA occurs on chromatin and does not mediate its degradation, but is important for the replication fork restart and homologous recombination at stalled replication forks.

Ubiquitin remnant profiling also enables global analyses of ubiquitin-linkage abundance and has been employed to reveal that UV light irradiation increases the cellular abundance of K6-linked ubiquitin chains (Povlsen et al., 2012; Elia et al., 2015a). Modification of proteins with K6-linked ubiquitylation by the ubiquitin ligase BRCA1 has been previously suggested to play a role in the cellular response to DNA damage (Wu-Baer et al., 2003, 2010; Morris and Solomon, 2004; Nishikawa et al., 2004).

Ubiquitin remnant profiling is a powerful approach for identification and quantification of ubiquitylation sites, however, several limitations should be considered when designing experiments: shotgun proteomics is biased towards more abundant peptide species and it is likely that ubiquitylation sites on low abundant, chromatin-associated proteins that often play an essential role in the regulation of DNA damage repair and signaling are missed. Also, the high amount of protein that is needed in order to achieve a satisfactory depth of the analysis hampers the application of this method for samples with limited quantity (e.g., primary cells). We envision that further improvements of MS instrumentation will facilitate the identification and quantification of low abundant ubiquitylation sites. Alternatively, targeted proteomics approaches can be employed to reproducibly quantify selected ubiquitylation sites across different experiments.

## Mapping Substrates of Ubiquitin-Modifying Enzymes

In addition to analyzing ubiquitin-dependent processes after DNA damage, an increasing number of studies have successfully employed ubiquitin remnant profiling and quantitative proteomics to identify substrates of ubiquitin ligases and deubiquitylating (DUBs) enzymes. In most studies the activity of the ubiquitin-modifying enzyme is inhibited using small molecules or the expression of the enzyme is down-regulated using knockdown or knockout approaches. Emanuele et al. (2011) employed ubiquitin remnant profiling and SILAC-based quantitative proteomics to quantify ubiquitylation sites in cells in which CRLs activity had been chemically inhibited by the NEDD8-activating enzyme inhibitor MLN4924. CRLs frequently target substrate proteins for degradation through the proteasome and therefore additional pre-treatment of cells with the proteasome inhibitor had been used to stabilize the proteins that are normally targeted for degradation by CRLs. The authors identified hundreds of ubiquitylation sites that decreased in abundance after CRL inhibition and demonstrated that NUSAP1, a protein involved in mitosis and DNA damage response, is a novel substrate of the Skp1, Cullin, F-box (SCF) Cyclin F (Emanuele et al., 2011). Elia et al. (2015a) employed a similar approach to demonstrate that 10% of UV light-induced ubiquitylation is dependent on CRLs and to show that SCF–Cyclin F ubiquitylates the double-stranded DNA exonuclease EXO1 after irradiation of cells with UV light.

Mutations in the substrate recognition domain of the CRL CUL3 adaptor speckle-type POZ protein (SPOP) are frequently found in primary prostate cancer (Barbieri et al., 2012). SPOP functions in DNA double strand break repair and SPOP mutations in prostate cancer are associated with genomic instability (Boysen et al., 2015). However, the identity of its substrates and whether or not cancer-associated mutations of SPOP affect the substrate landscape remained unclear. Theurillat et al. (2014) compared the abundance of ubiquitylation sites in immortalized prostate epithelial cells stably overexpressing wild type or mutant forms of SPOP. They were able to show that SPOP mutants lead to decreased ubiquitylation and impaired degradation of the chromatin remodeler DEK and thereby contribute to the oncogenic phenotype of prostate cancer cells.

Ubiquitin remnant profiling has also been employed to identify the substrates of the CRL adaptor cereblon (CRBN). Krönke et al. (2014) employed a chemical proteomics approach to show that lenalidomide, a thalidomide derivative used in treatment of multiple myeloma, binds to CRBN. Following up on this observation, they employed SILAC-based quantitative proteomics and ubiquitin remnant profiling to analyze alterations in ubiquitylation site abundance after treatment of MM1S multiple-myeloma cell line with lenalidomide. The authors demonstrated that lenalidomide regulates ubiquitylation and abundance of the transcription factors IKZF1 and IKZF3, and could further show that depletion of these proteins inhibited the growth of lenalidomide-sensitive multiple myeloma cell lines, while not having an effect on lenalidomide-insensitive cells (Krönke et al., 2014).

Furthermore, ubiquitin remnant profiling and inducible knockdown has been employed to identify DNA damage-inducible transcript 4 (DDIT4) as a novel substrate of the ubiquitin ligase HUWE1 that has been implicated in cancer development and DNA damage response (Thompson et al., 2014).

The above-mentioned studies demonstrate that ubiquitin remnant profiling is a powerful approach for identifying substrates of ubiquitin-modifying enzymes in the context of DNA damage signaling and disease. Development of novel specific small molecule inhibitors of ubiquitin-modifying enzymes as well as generation of knockdown/knockout cell lines will help to further decipher the complex relations between ubiquitin-modifying enzymes and their substrates.

## Investigating Ubiquitin Chain Topology

The role of K48-linked ubiquitylation in the degradation of proteins through the proteasome and the function of K63-linked ubiquitylation in cellular signaling are well established (Hochstrasser, 1995; Haglund and Dikic, 2005). The cellular functions of atypical ubiquitin chains formed through K6, K11, K27, K29, and K33 are largely unknown and tools for detection and enrichment of proteins modified with these atypical ubiquitin chains are missing.

The development of linkage-specific antibodies for M1-, K48-, and K63-linked ubiquitin chains facilitated the identification of substrates and functions of these ubiquitin chains (Newton et al., 2008; Matsumoto et al., 2010, 2012). Recently, high affinity binders (affimers) for K6, K33, and K48 ubiquitin chains have been generated and are commercially available; however, the application of these ubiquitin linkage-specific affimers for enrichment of proteins modified by specific ubiquitin chains still remains to be demonstrated. Efficient enrichment of proteins modified with a particular ubiquitin chain would enable their identification by LC–MS/MS and greatly help to deepen the understanding of the cellular functions of atypical ubiquitin chains (**Figure 1**).

In addition to antibodies and affimers, the identification of ubiquitin-binding domains (UBDs) that recognize specific type of ubiquitin chains allowed generation of engineered tandem ubiquitin-binding entities (TUBEs) that can be used as affinity matrix for enrichment of proteins modified with specific types of ubiquitin chains (Hjerpe et al., 2009; Sims et al., 2012). Recently, a K63-specific TUBE and SILAC-based quantitative proteomics have been used to compare protein ubiquitylation in wild type and UBC13 knockout HCT116 cells. Using this strategy, Thorslund et al. (2015) identified 371 proteins, several of which had been previously reported to be modified by K63-linked ubiquitylation. To complement these results, the same authors employed a similar approach for identification of proteins with increased K63-linked ubiquitylation after DNA damage induced by IR, thus identifying histone H1 as substrate of UBC13/RNF8 at DNA double strand breaks (Thorslund et al., 2015). Another study conducted in *Saccharomyces cerevisiae* employed a K63-specific TUBE for enrichment of K63-linked ubiquitylated proteins from wild type and ubiquitin K63R strain after oxidative stress induced by H_2_O_2._ The authors identified >100 proteins modified with K63-linked ubiquitin chains after treatment of cells with H_2_O_2_ and demonstrated that ribosomal proteins are dynamically modified by K63-linked ubiquitylation during the cellular response to H_2_O_2_ (Silva et al., 2015). Besides above mentioned TUBEs for K63-linked ubiquitin chains, TUBEs specifically binding to M1- and K48-linked ubiquitin chains have been generated (Trempe et al., 2005; Rahighi et al., 2009).

Another approach for analyzing ubiquitin chain topology on substrate proteins has been developed in the Komander lab: In Ubiquitin Chain Restriction Enzyme Analysis (UbiCRest), the relative SDS–PAGE mobility of investigated proteins before and after treatment with different linkage-specific DUBs is monitored to identify the type of ubiquitin chains on the protein (Hospenthal et al., 2015). Multiple DUBs from the human ovarian tumor (OTU) DUB family that display various degrees of specificities towards different ubiquitin linkage types have been identified and can be used for UbiCRest: For instance, OTUB1 specifically cleaves K48-, OTUD1 K63-, Cezanne K11-, and OTULIN M1-linked ubiquitylation, whereas OTUD3 displays specificity towards K6- and K11-linked ubiquitylation (Mevissen et al., 2013). A current limitation of this method is that DUBs might display various specificities towards ubiquitin chains linkages depending on the set-up of the assay and the concentration of the enzyme used, and the fact that specific DUBs for all types of ubiquitin chains have not been unambiguously identified. To date, UbiCRest was only employed to study the ubiquitin chain topology on single proteins; however, it might be possible to combine this method with MS to identify ubiquitin chain topologies on a proteome-wide scale.

## Conclusion

Development of methods for specific enrichment of ubiquitin remnant peptides and advances in high-resolution MS have enabled proteome-wide identification of ubiquitylation sites in cell lines and tissues. Furthermore, ubiquitin remnant profiling has been used for quantitative analysis of site-specific protein ubiquitylation after cellular perturbations, thereby providing a better understanding of the regulatory scope of ubiquitylation in different cellular processes, including the DNA damage response. Ubiquitin remnant profiling has also been successfully employed to identify substrates of ubiquitin-modifying enzymes, some of which have been implicated in the cellular response to DNA damage.

**FIGURE 2:**
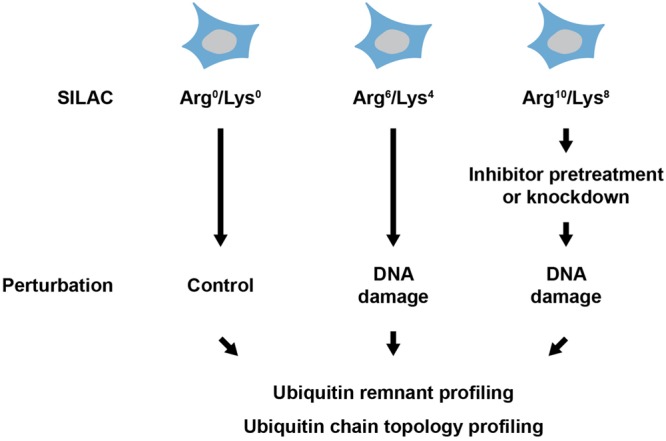
**Experimental strategy for analyzing DNA damage-associated ubiquitylation.** Cells are metabolically labeled using heavy isotope containing lysine and arginine (SILAC) and subsequently treated with a DNA damage-inducing agent. Pretreatment with an inhibitor or knockdown of ubiquitin-modifying enzyme can be performed before treatment with the DNA damage-inducing agent. Proteins are extracted from cells and ubiquitylation is quantitatively investigated using ubiquitin remnant profiling and/or ubiquitin chain topology profiling.

However, our understanding of the roles of ubiquitylation in the cellular DNA damage response is far from complete: little is known about the function of many of the dynamically modified ubiquitylation sites identified in ubiquitin remnant profiling studies. In addition, numerous ubiquitin-modifying enzymes have been implicated in the DNA damage response and for most of these enzymes the cellular substrate spectrum remains to be uncovered. Future studies employing ubiquitin remnant profiling and novel small molecule inhibitors or genetic knockdown/knockout approaches are likely to deepen the knowledge about the substrates and functions of these DNA damage-associated ubiquitin-modifying enzymes (**Figure 2**). Another major challenge lies in the investigation of the ubiquitin chain topology on proteins. In the last years, specific binders for M1-, K48- and K63-linked ubiquitin chains have been developed. Further development of tools for detection and enrichment of proteins modified with K6-, K11-, K27-, K29-, and K33-linked ubiquitin chains is essential to understand the cellular functions of atypical ubiquitylation. Probing the ubiquitin chain topology on proteins with DNA damage-regulated ubiquitylation sites will also help to understand the functions of ubiquitylation in the DNA damage response (**Figure 2**).

## Author Contributions

All authors listed, have made substantial, direct and intellectual contribution to the work, and approved it for publication.

## Conflict of Interest Statement

The authors declare that the research was conducted in the absence of any commercial or financial relationships that could be construed as a potential conflict of interest.
